# Update on Structure and Function of SH2 Domains: Mechanisms and Emerging Targeting Strategies

**DOI:** 10.3390/ijms26189060

**Published:** 2025-09-17

**Authors:** Moses M. Kasembeli, Jorge Rodas, David J. Tweardy

**Affiliations:** 1 Department of Infectious Disease, Infection Control and Employee Health, Division of Internal Medicine, University of Texas MD Anderson Cancer Center, Houston, TX 77030, USA; mmkasembeli@mdanderson.org; 2 Department of BioSciences, Rice University, Houston, TX 77005, USA; jorge.rodas@rice.edu; 3 Department of Molecular and Cellular Oncology, University of Texas MD Anderson Cancer Center, Houston, TX 77030, USA

**Keywords:** Src homology domain, phosphopeptide, inhibitor, docking, STAT3

## Abstract

The ultimate function of a protein is a summation of the activities of all its modules or domains. A major mechanism for regulating protein activity, besides modulation of its levels through translation or degradation, is covalent post-translational modification (PTM) of these modules, including phosphorylation and dephosphorylation of tyrosine, threonine, and/or serine residues. Phosphorylation is a fast, reversible, and highly specific mode of regulating protein function. Unlike proteins that are marked with other PTMs, phosphorylated proteins orchestrate an extensive network of protein interactions because of their ability to bind many protein partners. Protein phosphorylation is crucial for many cellular processes—signaling, transcription, and metabolism—because it precisely controls these processes in time and space. In this review, we will focus on signaling coordinated by tyrosine phosphorylation–dephosphorylation, specifically structural insights that govern the mechanism of recognition of phosphotyrosine (pY)-containing ligands by Src homology 2 (SH2) domains. We update the approaches used to target the SH2 domains and techniques applied in drug discovery, highlighting inhibitors that have reached clinical development.

## 1. Introduction

SH2 domains, approximately 100 amino acids long, are specialized modules that specifically bind phosphorylated tyrosine motifs. They form a crucial part of the protein–protein interaction network that is involved in many aspects of cellular function, including development, homeostasis, cytoskeletal rearrangement, and immune responses. The SH2 domain’s primary function in phosphotyrosine (pY) signaling networks is to induce proximity of protein tyrosine kinases (PTK) and protein tyrosine phosphatases (PTP) to specific substrates and signaling effectors by selectively recognizing proteins containing pY-peptide-binding motifs [1].

**Figure 1:**
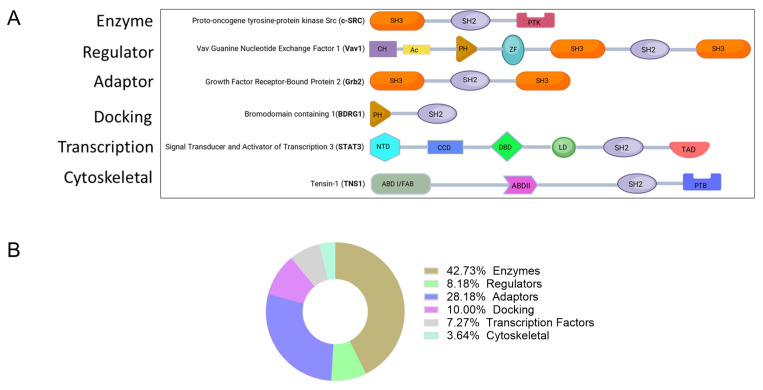
(**A**) Functional classes of SH2 domain-containing proteins. Most proteins within each classification contain multiple independently folding polypeptide modules shown as colored shaped objects (domains), i.e., SH2 domains along with other domains, including Src homology 3 (SH3) domains, protein tyrosine kinase (PTK) domains, actin-binding domains (ABD), focal-adhesion binding (FAB) domains, PTEN-like protein tyrosine phosphatase (PTP) domains, phosphotyrosine-binding (PTB) domains, calponin homology (CH) domains, acidic (Ac) domains, Dbl homology (DH) domains, pleckstrin homology (PH) domains, and C1, a zinc-finger like domain. The specific domains of STAT3 are delineated: N-terminal domain (NTD), coiled-coiled domain (CCD), linker domain (LD), and transactivation domain (TAD). (**B**) Percentage of SH2-containing proteins in each functional class.

Functionally diverse modular proteins contain SH2 domains; the human proteome includes roughly 110 such proteins. Researchers have shown that SH2 domains serve as modular regulators in multidomain proteins (Figure 1), broadly classifiable into several groups, including enzymes, signaling regulators, adapter proteins, docking proteins, transcription factors, and cytoskeleton proteins [2]; [Figure 1 and Table 1]. SH2 domains not only facilitate protein–protein interactions, but they also bind other biologically active molecules such as lipids. Lipids are a major component of the plasma membrane and play a crucial role in cell signaling by associating with phospholipid-binding domains [3].

**Figure 2:**
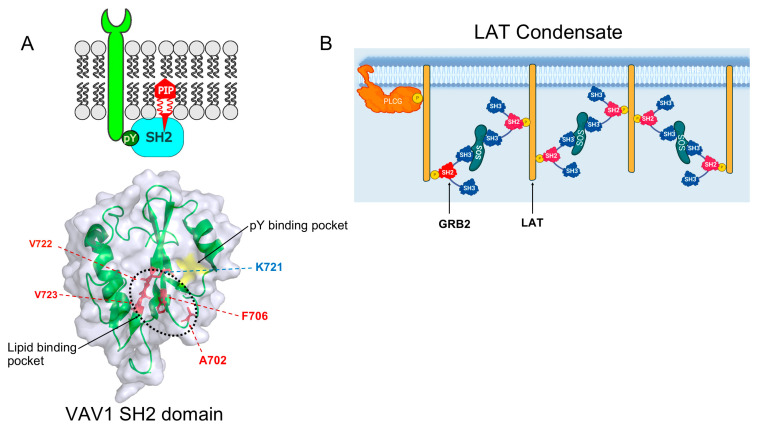
(**A**) Schema of a lipid interaction with an SH2 domain-containing protein. The SH2 domain of VAV2 protein (Protein Data Bank (PDB): 2LNX) [8] showing the lipid-binding residues labeled blue cationic amino acid—Lys, flanked by hydrophobic amino acids in red, forming a region (black dotted line) distinct from the pY-binding pocket. Highlighted in yellow is the phosphotyrosine-binding pocket. (**B**) An example of the role of SH2-containing proteins in the formation of phase-separated condensates. Phosphorylation events trigger PLCg, GRB2, SOS, and Linker for Activation of T cells (LAT) complex assembly, leading to condensate formation during T-cell activation.

Recent research shows that nearly 75% of SH2 domains interact with lipid molecules in the membrane, with a tendency towards phosphatidylinositol-4,5-bisphosphate (PIP2) or phosphatidylinositol-3,4,5-trisphosphate (PIP3) [4] (Table 2). Studies have identified cationic regions in the SH2 domain that are close to the pY-binding pocket as lipid-binding sites, which are usually flanked by aromatic or hydrophobic amino acid side chains (Figure 2A). Lipid–SH2 domain interactions modulate the cell signaling of SH2-containing proteins. For example, the PIP3 binding activity of the TNS2 SH2 domain is important in regulating phosphorylation of insulin receptor substrate-1 (IRS-1) in the insulin signaling pathway [5]. VAV, LAC, and ZAP70 are other examples that back the idea that lipid binding by SH2 domains is vital for the enzymatic activity and scaffolding roles of SH2-containing proteins [6]. Studies have also shown that many disease-causing mutations in SH2 domains are localized within the lipid-binding pocket of SH2 domains [6]. New research indicates that targeting lipid binding in SH2 domain-containing kinases may offer a promising avenue for the development of new small-molecule drugs. Cologna and colleagues have successfully developed nonlipidic inhibitors of Syk kinase. The data indicate that nonlipidic small molecules are capable of specific and potent inhibition of lipid protein inhibitors (LPI). This approach could produce potent, selective, and resistance-resistant inhibitors for various other kinases possessing the SH2 domain [7].

**Table 2:** SH2 domain-containing proteins that bind lipids with proven functional activities.

Protein Name	Function of Lipid Association	Lipid Moiety	Ref
**SYK**	PIP3-dependent membrane binding is required for the activation of SYK scaffolding function, leading to noncatalytic activation of STAT3/5	PIP3	[7]
**ZAP70**	Lipids are essential for facilitating and sustenance of ZAP70 Interactions with TCR-ζ n	PIP3	[7]
**LCK**	Modulates the interaction of LCK with its binding partners in the TCR signaling complex	PIP2, PIP3	[9]
**ABL**	Membrane recruitment and Modulation of Abl activity	PIP2 interaction	[4,10]
**VAV2**	Modulates the interaction of VAV2 with membrane receptors, e.g., EphA2	PIP2, PIP3	[11]
**C1-Ten/Tensin2**	Regulation of Abl activity and the phosphorylation of IRS-1 in insulin signaling pathways	PIP3	[5]

Proteins with SH2 domains have increasingly been linked to the formation of intracellular condensates via protein phase separation (PPS). Multivalent interactions, such as those associated with modules such as SH2 and SH3 domain interactions, drive condensate formation [12]. Post-translational modifications, including phosphorylation, modulate the assembly and disassembly of condensates. Liquid–liquid phase separation (LLPS), associated with membrane receptors, forms important signaling entities that are controlled by phosphorylation (Figure 2B; Table 3).

Studies have shown that interactions among GRB2, Gads, and the LAT receptor contribute to LLPS formation, enhancing T-cell receptor signaling [17]. In podocyte kidney cells, LLPS is thought to increase the ability of adapter NCK to promote N-WASP–Arp2/3–mediated actin polymerization by increasing the membrane dwell time of N-WASP and Arp2/3 complexes [18]. In this review, we summarize the primary cellular function of the SH2 domain, followed by a focused discussion of structural and functional insights into its binding mechanism, specifically addressing key structural determinants of phosphotyrosine (pY)-containing ligand recognition. Building upon existing foundational knowledge, this review offers an updated emerging biochemical, biophysical, and computational methodologies for investigating ligand binding, structural dynamics, and the identification of small-molecule inhibitors targeting SH2 domain-mediated protein interactions. With increasing efforts to study both canonical and non-canonical roles of the SH2 domains in signaling events, one could envisage the discovery of new mechanisms of disease that provide a foundation for novel therapeutic agents.

## 2. SH2 Domain Structure and Binding to pY-Peptide Ligands

### 2.1. SH2 Domain Structure

The structure of SH2 domains has been the subject of intense study since their discovery by Pawson and colleagues [19] in 1986. To date, the structures of 70 SH2 domains have been experimentally solved with varying degrees of resolution. Despite having some family members with as little as ~15% pairwise sequence identity, all SH2 domains assume nearly identical folds [20,21]. Remarkably, SH2 domains show very little divergence within their three-dimensional folds and function, suggesting that these folds have evolved almost exclusively to bind pY-peptide motifs.

**Figure 3:**
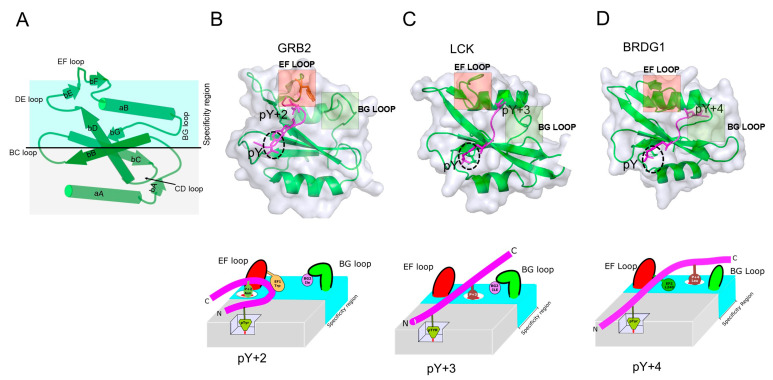
(**A**) A cartoon showing the configuration of the secondary structural elements of SH2 domains and their binding modes to pY-peptide ligands. SH2 domains are shown in (**B**) GRB2 (PDB:1JYR) [24], (**C**) LCK (PDB:1LCJ) [25], and (**D**) BRDG1 (PDB:3MAZ) [26], representing three major phosphopeptide-binding modes of SH2 domains with peptide specificity residues at pY+2 for GRB2, with its pY-peptide adopting a beta turn, while the pY-peptides with specificity residues at pY+3 and pY+4 that bind LCK and BRDG, respectively, assume a linear configuration. The black dotted circle denotes the phosphate binding site. Below each structure is a cartoon representation of each binding mode, highlighting the loop structures that govern peptide specificity [26].

**Figure 4:**
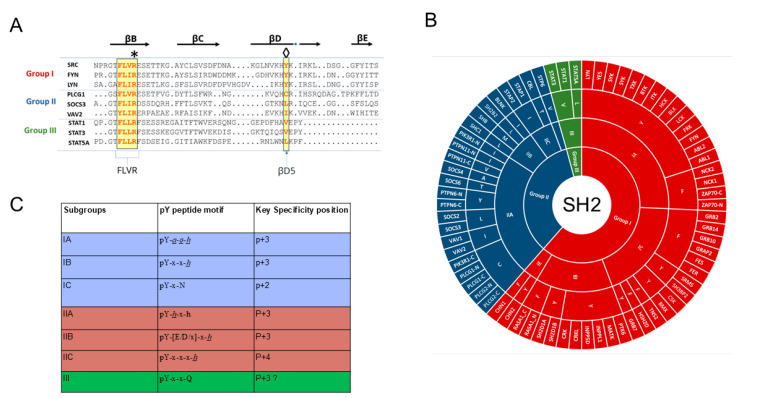
SH2 domain classification: (**A**) Representative sequences of SH2 domain proteins from each group showing the FLVR region; the asterisk (*) denotes the crucial arginine residue responsible for phosphate binding and the location at position 5 (βD5) (◊), whose identity is central to the classification scheme shown in B. Top strands represent beta strands B, C, D, and E. (**B**) A four-layer color-coded sunburst plot showing the classification of SH2 domains, whose structures have been solved (for a complete list see reference) [27]. There are three SH2 domain categories or layers: the first layer consists of red, blue, and green groups corresponding to groups I, II, and III, respectively. This is followed by subgroups in layer 2, classified by amino acid at βD5 and binding specificity at the pY+ position. Layer 3 is a residue at position βD5, denoted in panel A, whose identity specifies the group’s category (Group 1: Y/F; 2: I/L/V/C/M; and 3: E/Q/L. (**C**) Table showing subgroups with known pY-peptide motifs that bind the SH2 domain and position of specificity residues. (h = hydrophobic amino acid; a = acidic amino acid; x = any amino acid).

The basic structure of SH2 domains is a “sandwich” consisting of a three-stranded antiparallel beta-sheet that is flanked on each side by an alpha helix—αA-βB-βC-βD-αB. The majority of the SH2 domains contain extra secondary structural elements, including beta strands A, E, F, and G, to make a total of seven motifs. Structurally, the N-terminal region of the SH2 domain is highly conserved, while the C-terminal region is variable (Figure 3A). The N-terminal region of the SH2 domain contains a deep pocket that is located within the βB strand that binds the phosphate moiety; this pocket harbors the invariable arginine (R) at position 5 (βB5), which is a part of the FLVR motif found in all SH2 domains except for three SH2 domains, which have an aromatic residue at this location instead [22]; [Figure 3B,C and Figure 4A]. This arginine directly binds to the pY residue within peptide ligands through a salt bridge. The C-terminal region contains b stands E, F, and G, which are observed in most but not all SH2 domains. Interspaced between each SH2 structural element are intervening unstructured regions (loops). The length of the CD-loop varies depending on the category of protein family. SH2 domains of enzymatic proteins tend to have longer loops as compared to non-enzymatic proteins such as STATs [23]. The 3D conformation and relative spatial orientations of the loops also provide an additional level of diversity, as loops within the SH2 domains play an important role in phosphopeptide binding. The EF loop, which joins the β-strands E and F, and the BG loop, which joins the α-helix B and β-strand G (Figure 3A–D), especially play crucial roles in determining binding selectivity by controlling access to the ligand specificity pockets.

Structurally, SH2 domains can be divided into two major subgroups—the STAT type and SRC type. STAT-type SH2 domains are distinct in that they lack the βE and βF strands as well as the C-terminal adjoining loop. The αB helix is also split into two helices. This disparity is likely an adaptation that facilitates dimerization, a critical step in STAT-mediated transcriptional regulation, reflecting the ancestral function of SH2 domain-containing proteins that predate animal multicellularity, as observed in *Dictyostelium,* which employs SH2 domain/phosphotyrosine signaling for transcriptional regulation [20,23,28].

### 2.2. Specificity Determinants of SH2 Domain Binding to pY-Peptides

Approximately 40% of the interactions between proteins involve proteins that are bound to peptides or proteins that are bound to peptide-like sequences that are located within unstructured regions of other proteins [29]. These interactions are characterized by a fast off-rate to allow for specific but short-lived interactions, which is a defining characteristic of most cell signaling mediator interactions. SH2 domain binding is characterized by a combination of high specificity toward cognate pY ligands with moderate binding affinity (Kd) 0.1–10 µM [30]. This is consistent with protein–protein interactions that are geared toward orchestrating short-lived events to assure responsive intracellular signaling [31].

The balance between binding affinity and specificity is achieved via a two-pronged binding mechanism. The first prong is the pY residue, and the second one involves residues immediately C-terminal to the pY that determine specificity for the pY-peptide. Residues in the pY recognition site of SH2 domains are located within αA, βB, βD, and the BC loop of the SH2 domain. The second prong (specificity residue) of the pY-peptide binds to a hydrophobic region in the SH2 domain formed by strands C and D (Figure 3A). Structural data show that most SH2 domains recognize the pY of cognate peptides in a similar fashion. However, despite the overall structural similarity of SH2 domains, they bind pY-peptides selectively, with stringent primary sequence requirements. Peptide-specific interactions are mediated primarily by the peptide residues’ C-terminal to the pY residue.

The canonical amino acid sequence in peptides that are recognized by SH2 domains is Y1-X2-X3-X4, where Y is the phosphorylatable tyrosine and X is any amino acid located at the +1, +2, or +3 residues C-terminal of Y. The structural data show that the hydrophobic region on the SH2 domain allows for distinct interactions that endow specificity for peptide binding. An absence of phosphorylation on a cognate peptide almost completely abrogates binding, suggesting that the energetics of binding are derived mainly from the key interaction with the arginine within the βB5 that is buried deep in the structure, and this is only accessible to the longer R group within pY as opposed to pS or pT. In the presence of the pY, peptide binding specificity is dependent on the residues downstream of the phosphorylation site. The underlying basis for peptide selectivity has been the subject of extensive investigations that have spurred various classification schemes. To delineate the specificity of the SH2 domains, the sequence selectivity of various SH2 domains has been analyzed utilizing pY-peptide libraries. Oriented peptide array library (OPAL) studies have examined preferences of SH2 domains for residues at various positions within the pY-peptide relative to the pY [27]. Generally, the specificity preference of the majority of SH2 domains falls into three groups, i.e., selectivity for a specific amino acid at the pY+2 position, the pY+3 position, or the pY+4 position (Figure 4A,B). The classification of SH2 domains is based on the identity of the residue at position beta strand D residue5 (βD5). Therefore, we can broadly categorize human SH2 domains into three primary groups—group I, II, and III—where group I contains either Y or F at the βD5 position; group II has a non-aromatic, non-polar residue (I/L/V/C or M); while group III (mostly STAT proteins) has a hydrophobic group (V or L) [27] (Figure 4A–C). In addition, a further sub-classification has been presented within each of the three groups; these subclasses use amino acid preferences at the Y +1, +2, +3, or +4 positions of the pY-peptide as classifiers in addition to residues at βD5.

### 2.3. Contextual Peptide Specificity

Despite assays using peptide libraries having success in identifying amino acid preference at positions flanking the pY residue, they do little to explain the secondary effects that undoubtedly make significant contributions to the specificity of SH2 domains toward cognate pY-peptides. To overcome this limitation, others have looked at the effect of specific amino acid residues in the context of other residues within the primary sequence of the pY-peptide (contextual effects) and have shown that the peptide sequence context is a major determinant for SH2 domain selectivity.

For example, an interesting conundrum is why SH2 domains that recognize conserved core binding motifs, such as the pY-x-x-P motif [32,33], have a preference for only a small fraction of peptides containing this motif. Contextual peptide specificity provides a plausible explanation for the differential selectivity, and it has been shown that neighboring amino acid positions within the peptide sequence affect one another, such that depending on the hydrophobic binding surface of the SH2 domain, residues immediately of the C-terminal of pY on the peptide ligand will either lead to favorable contributions to the overall binding energy (permissive) or unfavorable contributions (non-permissive) [32].

### 2.4. Contribution of Loop Regions on SH2 Domain/pY-Peptide Selectivity

How does the structural surface of the SH2 domain influence peptide binding and selectivity? Comparisons among the three major representative motif recognition groups (P+2, +3, and +4) of the SH2 domains reveal the critical roles that the EF and BG loops play in determining pY-peptide selectivity. The loops adopt different structural configurations that control access to binding sites and influence the binding conformation of peptide ligands. Despite belonging to the same superfamily defined by a conserved structural fold, some SH2 domains exhibit notable variations within their EF and BG loops. In work by Nash et.al, the authors analyzed structures of different SH2 domain groups and their interaction preferences; they found that loops and the amino acid side chains within them act as plugs that influence the path of the pY-peptide across the SH2-peptide binding surface. This, in turn, dictates the selectivity requirements of residues immediately after the pY [26,32].

Several studies offer insights into how loops in the SH2 domain govern selectivity. Examples include the SH2 domains of the Src family of kinases, for example, LCK (Figure 3), in which a change in position 1 of the EF loop (EF1) from Thr to Trp effectively switched selectivity of the LCK SH2 domain from a hydrophobic amino acid at pY+3 to an Asn at the pY+2. This switch in binding selectivity is similar to the GRB2 and BRDG1 SH2 domains that bind hydrophobic residues at P+4: both selectivities can be converted to pY-peptides with tryptophan at position P+3 (Figure 3). These examples highlight the important role that loop structures of SH2 domains play in governing the domain’s selectivity for pY-peptides. This knowledge is essential in developing effective and specific inhibitors of SH2 domain-containing proteins.

## 3. Plasticity in SH2 Domain Binding and Allostery

The prevailing mechanism of SH2 domain/pY-peptide binding has been the two-prong binding model. This static key and lock mechanism of binding, however, is not adequate in some instances for describing SH2 domain binding to pY-peptides. It is known that for every protein, there is more than one conformational state available, and proteins exist as an ensemble of conformations. Understanding the dynamics of protein–protein interactions benefit from considering molecular cooperativity, a phenomenon that underlies different modes of molecular recognition, such as induced fit or allosteric binding. Recent structural studies and computational modeling suggest a more complex mode of binding that is not fully captured by the key and lock mechanism; For example, HSQC (Heteronuclear Single Quantum Coherence)-generated chemical shift perturbation (CSP) data comparing free and bound N-SH2 domain of PI3K reveal conformational changes throughout the whole domain. A combined strategy involving site-directed mutagenesis and NMR experiments revealed the structural plasticity of the N-SH2 domain. Kinetic data show that an allosteric network involving long-distance perturbations appears to contribute to the recognition of the methionine residue at the pY+3 position of N-SH2 domain peptide ligands [34]. Studies that have compared two SH2 domains—phospholipase Cy1 and Syp phosphatase—showed differences in binding mechanisms despite having structural features that would suggest similar modes of binding; these studies found that differences in side-chain dynamics of residues within the hydrophobic pocket could explain these differences in binding mechanisms. NMR relaxation experiments that looked at nanosecond–picosecond (ns-ps) time scale side-chain dynamics suggested a correlation between binding affinity and dynamics [35].

The linker for the activation of T cells (LAT)—a protein with multiple pY modifications that binds to GRB2—is another intriguing example that highlights the role of protein dynamics in SH2 binding. It has been observed that the binding affinity of pY-LAT to GRB2 increases with the increasing number of pY-modified sites beyond the specific GRB2 pY-peptide ligand within LAT [36], raising the question of how GRB2 senses pY sites remote to its binding site. Recent studies have evaluated the dynamic properties of the GRB2-SH2 domain and how it influences the binding of pY-peptides. GRB2-SH2 has two dynamically independent subdomains—subdomain I contains residues that are involved in fast-exchange rate recognition of the pY residue, and subdomain II contains residues that are involved in intermediate-exchange rate recognition of the pY + 2 specificity site. The chemical exchange that was observed by NMR suggests that pY binding by GRB2 proceeds through an intermediate or encounter complex, which involves an ensemble of GRB2 orientations that are influenced by the status of remote pY sites, leading to the formation of a final bound complex [37].

Protein dynamics and conformational transitions govern the SH2 domain allosteric properties. Allosteric regulation is a regular feature in many proteins that contain an SH2 domain. Recent data suggest the prevalence of long-range communications within many modular proteins that contain SH2 domains [38,39,40,41]. For example, mutations in STAT3 support evidence of long-range interdomain allosteric communication that is propagated from relatively remote domains to impact pY-peptide binding. Mutations within the coiled-coiled domain (D170A) and STAT3 linker domain (I586F) have been shown to severely impact its transcriptional activation by disrupting the binding of its SH2 to pY-peptide [42]. Molecular dynamic simulations and NMR data show that the linker domain of STAT3 is critical for the interdomain transmission of conformational changes that are coupled to intra-domain allosteric dynamics of its SH2 domain [43].

Many of the intriguing and unconventional modes of SH2/pY-peptide binding can be attributed to long-range inter- or intra-domain protein dynamic effects on SH2 domains. For example, mutation W165C, located within the regulatory domain of the ZAP-70 kinase, causes autoimmune arthritis in SKG mice through dysregulated T-cell signaling. The two structurally independent SH2 domains of ZAP-70 bind cooperatively to the double pY-peptide motifs within the immunoreceptor tyrosine activation motif (ITAM) of the T-cell receptor (TCR). The binding of the N-SH2 domain is dependent on the initial interaction of the C-terminal SH2 (C-SH2) domain. Molecular dynamics (MD) simulations, NMR spectroscopy, and biochemical analysis revealed an allosteric network of residues located between the two SH2 domains that included W165, which is necessary for this dependence; mutation W165C disrupted it and reduced ZAP-70 binding to the TCR [44].

A unique secondary site (exosite) that is located on the side of the SOCS2 SH2 domain, opposite to its pY-peptide binding site, has been shown to lower the off-rate of bound pY-peptide ligands, leading to an increase in the binding affinity, providing yet another example that highlights the significance of intra-domain allosteric communications to SH2 domain pY-peptide binding.

Binding models for SH2 domains have often been limited to the static snapshots of protein–phosphopeptide complexes presented by crystal structures. The work highlighted above clearly indicates that conformational plasticity has a crucial role in modulating the binding affinity and selectivity of SH2 domains for p-Y-peptide ligands. A deeper understanding of SH2 domain protein dynamics and allosteric regulation may inform efforts to rationally design modulators of SH2 domain-containing proteins.

## 4. Emerging Trends in Modeling SH2 Domain/pY-Peptide Binding

While experimentally generated three-dimensional (3D) structures of proteins and their complexes provide invaluable mechanistic insights into how proteins function at the atomic level, the information they provide often is incomplete. For example, structures resolved using X-ray crystallography data sometimes diverge from experimental data by up to 20% [45,46]. Proteins are dynamic entities, not static, as frequently presented by crystal structures; static structures do not always explain the complexities of protein–protein interactions [47]. Popular conformational search methods, such as Monte Carlo (MC) and MD, are being used increasingly to complement static data and have proved useful in elucidating complex structural phenomena with a high resolution and providing information that would otherwise not be garnered from static structures. MD simulations based on high-resolution static structures have been used to calculate the binding energies of SH2 domains, to characterize protein–protein interactions, and to identify new interacting partners [48].

### 4.1. Search Methods for Computational Analysis of Peptide Binding Conformations

To date, only 10 structures of SH2 domains in a complex with their cognate pY-peptide have been resolved at atomic resolution. To delineate additional SH2/pY-peptide structures that are important to major signaling pathways, prediction methods are necessary to fill in the information that is either missing or poorly resolved in static structures. MD simulations have been used in these instances to study protein–protein interactions at the atomic level. For example, the molecular mechanics Poisson−Boltzmann surface area (MM-PBSA), which involves the postprocessing of several snapshots of a complex in implicit solvents that are extracted from MD simulation, has been used to accurately estimate binding free energies of the pY-peptide bound to the GRB2-SH2 domain. A comparison of an MD simulation with SPR experimental data of nine different pY-peptides bound to GRB2 found that the MM-PBSA continuum solvent method could accurately determine pY-peptide specificity. Moreover, the calculated free energies showed a significant correlation (R = 0.92), demonstrating the potential utility of this method for SH2 domain ligand screening [49]. Other strategies employing force fields that are based on empirical data to predict protein energetics, such as the Fold-X algorithm, have also been used successfully to determine SH2/pY-peptide binding specificities that agree with results from experimental methods using peptide libraries. Overall, these computational methods may be instrumental in uncovering underlying binding mechanisms of different SH2 domains with implications for designing new therapeutics.

Large structural rearrangements and allosteric regulation cannot be characterized by X-ray crystallography alone [50]; improvements in MD methodologies have yielded results that challenge well-accepted models of SH2 domain interactions based on crystal structures. For example, previous crystallographic data suggested that the binding cleft of N-SH2 of SHP2 serves as the crucial “allosteric switch” that is involved in SHP2 activation. However, MD simulations show that the dynamics of the loops that constitute the binding cleft had no effect on the free energy of activation. Instead, the allosteric switch results from the opening of the central beta-sheet of the N-SH2 [51]. Additional studies using MD simulations and NMR spectroscopy confirmed that apo N-SH2 in solution primarily adopts a conformation with a fully zipped central β-sheet, which is promoted by the binding of phosphopeptide. Additional studies highlight the importance of complementing experimental data with in silico conformational modeling. For example, high-resolution crystal structures suggest that the SH2 domain of Fer binds to its cognate pY-peptide in distinctly different ways and, in one instance, adopting a type I β-turn conformation and, in another instance, a fully extended conformation [52,53]. MD simulation is the tool of choice for describing structural flexibility of proteins. Nachman et. al. used simulated annealing (SA) to sample the conformational space of pY-containing peptides that are bound with the Src SH2 domain. In addition to an extended conformation, pY-peptides assume other conformations, including an a-helix and b-turn, which is similar to the confirmations of pY-peptide bound to the GRB2 or STAT3-SH2 domain [54,55]. Overall, these results support the use of computational tools to study pY-peptide interactions to optimize drug discovery efforts.

### 4.2. Machine Learning and Neural Networks

Accurate models of SH2 domain/pY-peptide binding within biological complexes are necessary to understand key signaling networks involving pY PTMs. There are approximately 5.5 million possible SH2/pY-peptide interactions in the human proteome, of which only 2% have been identified through various techniques, including recent high-throughput analysis. There is a crucial need to fill this informational gap by using computational methods. However, this effort has been hampered at times by a lack of good training data sets due to an imbalance in the data, i.e., lack of negative data, as training models require both positive and negative datasets. Additionally, in some cases, there is limited agreement on positive interactions between data generated by different research groups and platforms. The use of these datasets for predicting SH2/pY-peptide interactions has been shown to lead to sub-optimal results, as seen in methods based on position-specific matrix scoring PSSM [56]. Several groups have developed statistical approaches in attempts to reconcile inconsistencies between datasets that have had some success in reducing the false-negative rate and increasing the amount of useful data that are available to use in prediction models [57,58].

To overcome some of the issues posed by the poor quality of available pY-peptide binding data in predictive models, Kundu et al. used a machine learning approach based on an efficient semi-supervised learning technique that successfully addressed the imbalance in the data between confirmed interactions (positive data) and empirically proven non-interactions. This approach was able to make genome-wide prediction of human SH2-peptide binding of 51 SH2 domains [56,59]. While significant progress has been made in understanding SH2/pY-peptide interactions, further efforts are needed to globally map the SH2/pY-peptide interactome. With the advent of new, powerful AI-based deep learning frameworks such as Alphafold3, it will be possible to use them in a high-throughput manner to more comprehensively understand pY-modified proteins and their interactions with SH2 binding partners.

## 5. Categories of SH2 Domain Inhibitors

### 5.1. Peptidomimetics

Early proof-of-principle studies showed that a peptide mimetic of the SH2 binding interface prevented formation of the active complexes, leading to loss of biological activity [60,61,62]. The major drawbacks of this approach for a long time had been low plasma membrane permeability in addition to the high lability of phosphate groups. Improvement of peptide delivery mechanisms has made this approach viable. Several groups have successfully used a peptidomimetic strategy to disrupt SH2 domain interactions. For instance, Mandal et al. capped the phosphate oxygen atoms of a high-affinity STAT3 binding phosphopeptide with an enzyme-cleavable group—pivaloyloxymethyl (POM)—and replaced the phosphate with a phosphonodifluoromethyl moiety to create phosphatase-stable, cell-permeable peptidomimetic prodrugs that inhibited the phosphorylation of STAT3 in breast cancer cell lines [63]. A similar approach was used by the same group to inhibit Tyr641 phosphorylation and transcriptional activity of STAT6 [64]. Other groups have demonstrated that the cell-permeable peptides, such as STAT-6-IP, inhibited OVA-induced production of Th2 cytokines IL-4 and IL-13 in vitro [65]. Recent data suggest that disruptions of the protein–protein interaction (PPI) interface of the SHP2 SH2 domain, rather than its enzymatic activity, may be a more effective approach to inhibiting SHP2 biological activity in the setting of mutations that are linked to childhood myelodysplastic syndromes and various forms of leukemias. A peptide derived from insulin receptor substrate 1 (IRS-1) that has nanomolar affinity for the N-SH2 domain of SHP2 was shown to reverse the effects in zebrafish of the D61G activating mutation of SHP2 [66].

### 5.2. Covalent Inhibitors

**Figure 5:**
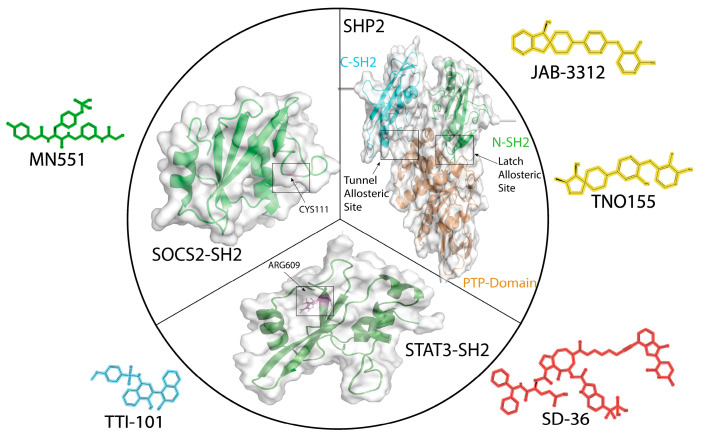
Structures of three proteins whose SH2 domains were targeted by small molecules using strategies described in this review. MN551 (green) is a covalent inhibitor that attaches to Cys111 in the EF loop within the SH2 domain of SOCS2. JAB-3312 and TNO155 (yellow) are allosteric inhibitors of SHP2 that bind to its latch site or tunnel site, respectively. TTI-101 (blue) is a competitive inhibitor that targets the phosphotyrosine (pY)-peptide binding site of STAT3. SD-36 (red) is a PROTAC, one portion of which binds to the pY-peptide binding site of STAT3, while the other portion binds to cerebron, a key component of the E3 ubiquitin ligase complex.

Despite the well-known risk of off-target effects, the development of irreversible inhibitors is gaining interest for targeting “undruggable” PPI interfaces. These molecules are designed to block interactions by covalently bonding to reactive amino acids at critical protein sites on the protein’s surfaces. Covalent inhibitors provide sustained inhibition for the lifetime of the protein target, may be effective at a reduced dosage compared to competitive inhibitors, and potentially overcome drug resistance mechanisms [67]. Early studies suggested that this approach may be feasible for SH2 domain-containing proteins. For example, one of the earliest small molecules discovered that targeted the SH2 domain of STAT3, STATIC, acts via a Michael addition reaction with a critically positioned cysteine within the SH2 domain. More recently, Ramachandran et al. developed NM551 in which a chloroacetamide group is added to a peptidomimetic to serve as a covalent warhead to target a cysteine residue (Cys111) in SOCS2 [68]. (Figure 5) Other examples include results from HTP screens by Beshore et al. that yielded potent covalent inhibitors of the SYK SH2 domain that disrupt its interaction with the FCER1G peptide [69] and Deng et al., who demonstrated the feasibility of targeting the STAT3-SH2 domain with the boronic acid group, which has reversible covalent binding properties that lead to enhanced binding affinity with STAT3 SH2 domain [70]. With the development of new warhead chemical entities, covalent inhibitors aimed at targeting SH2 domain-containing proteins will expand the chemical space beyond simple competitive inhibitors.

### 5.3. Proteolysis Targeting Chimeras (PROTAC)

Targeted protein degradation (TPD) is a promising, innovative therapeutic approach that employs chemically induced protein complexation to decrease protein abundance; examples include heterobifunctional PROTAC and low-molecular-weight, drug-like molecular glues, both of which mediate proximity-induced protein degradation by recruiting target protein to E3 ligases. Unlike traditional small-molecule inhibitors that act by binding either competitively or non-competitively to a pocket that is crucial for the target protein’s function, PROTAC and molecular glues have the advantage that they do not require identifying a functional binding pocket on a target protein. Thus, TPD has a greater chance of targeting otherwise intractable or “undruggable” proteins compared to conventional approaches. Furthermore, unlike small-molecule competitive inhibitors that necessitate high-affinity binding for pharmacological impact, PROTACs remain effective even at lower binding affinities. This approach has been tested in several proteins with SH2 domains. An early example is SD-36, a bifunctional molecule with one end consisting of a phosphomimetic moiety that binds to the pY-peptide binding site within the STAT3 SH2 domain, while the other end consists of a cereblon ligand that interacts with CRBN/cullin 4A. SD-36 reduced levels of total STAT3 within cells, induced apoptosis of acute myeloid leukemia and anaplastic large-cell lymphoma cell lines, and achieved tumor regression of cell line xenografts in mice. Other examples of TPD approaches used to target SH2 domain-containing proteins include SHP099 that targets SHP2 and PROTAC degrader AK-2292 that targets STAT5 [71,72]. Of note, a phase 1 trial of KT-333, a STAT3 PROTAC, to treat patients with relapsed or refractory lymphomas, large granular lymphocytic leukemia, and solid tumors, has been completed but has not been published [73]; The company developing KT-333, Kymera, is looking to partner before proceeding to phase 2. Another PROTAC program within Kymera that appears even more promising targets STAT6 [74]. A note of caution with TPD approaches, however, is that many SH2 domain-containing proteins are multifunctional. Unlike small-molecule chemical probes that selectively target a single function of a protein, TPD approaches target the entire protein and all its functions, potentially causing on-target toxicities.

### 5.4. Competitive Orthosteric and Allosteric Small-Molecule Inhibitors

As discussed above, their low activity, poor stability, and poor cell penetration typically limit peptide-based inhibitors. Consequently, small molecules that target the phosphopeptide binding sites have attracted significant attention. The SH2 domain’s specific natural ligands (pY-peptides) are ideal for competitive inhibition assays that are frequently used to directly screen libraries of small molecules for the identification of competitive orthosteric inhibitors, as well as allosteric inhibitors, of pY-peptide binding, and have resulted in the discovery of many molecules currently under preclinical testing. However, despite prodigious efforts, very few SH2 domain inhibitors have reached the clinical development phase. The complexity and dynamic nature of SH2-containing proteins, coupled with limited detailed structural data on crucial interaction sites, may be responsible for this lack of progress. Although highly conserved, the orthosteric ligand-binding sites within SH2 domains may pose challenges because of unexpected conformational dynamics in the region responsible for pY-peptide binding. Consequently, appropriately sized drug-binding pockets (cryptic sites) might not be readily discernible in the structure of some proteins, either in their unbound form or when bound to their endogenous ligands. Researchers have developed a variety of experimental and computational tools, such as combining molecular dynamics with fragment docking and machine learning approaches, to identify cryptic sites. These sites may not be evident in the unbound protein but form upon ligand or chemical probe binding and can reveal novel targetable drug sites [34,75,76,77]. To date, orthostatic inhibitors of STAT3 and allosteric inhibitors of SHP2 protein are the most advanced inhibitors currently in clinical development (Figure 5).

Other structure-based drug discovery efforts have identified inhibitors and inactivators of SH2 domain-containing proteins that are currently in clinical development. (Table 4). These inhibitors are comprised mainly of small molecules that are discovered from synthetic chemical or virtual libraries through structure-based design and molecular screens that are aided by structural data [78,79,80].

## 6. Small-Molecule Screening and Lead Compound Identification of SH2 Inhibitors

### 6.1. Structure-Based and High-Throughput Screens

The earlier methods of SH2 domain inhibitor lead discovery involved techniques, such as cell-based screens, enzymatic assays, and fluorescence polarization (FP) assays. X-ray crystallography and NMR have been the major techniques that have supported structure-based screens and high-throughput screens (HTS) that provided data for the rational design of SH2 targeting compounds. These techniques have also been instrumental as complementary tools for confirmation of other biophysical screens targeting the SH2 pY-binding pocket, such as FP. In recent years, information gained from advances in structural biology has sped up progress in screening technologies, including computational, biophysical, and cell-based assays, which have resulted in a significant improvement in hit rates with fewer false positives. Data obtained from functional assays show that structure-guided approaches have yielded molecules that are effective at disrupting complexes of SH2 domain proteins that form through pY-peptide interactions [81]. Fragment-based drug discovery (FBDD) is another technique that employs biophysical and biochemical methods such as nuclear magnetic resonance spectroscopy (NMR), X-ray crystallography, and surface-plasmon resonance (SPR) to screen low-molecular-weight molecules or “fragments” for binding to a specific target. These “fragment” hits serve as starting points for the generation of leads. In a recent example, Day et al. used structure-guided optimization combined with solvent mapping MD to discover compound 28 that binds to a known allosteric tunnel binding site (Tunnel Site) that is active in the low-micromolar range in vivo [82]. New multiplexing methodologies, such as DNA-encoded libraries (DEL), which allow for multiple screens to be performed at once in a single binding assay against a target protein, have recently technologically boosted high-throughput screens (HTS). This strategy has been adopted by different research groups to develop inhibitors that target the SH2 domains of different proteins [83,84].

### 6.2. Computer-Aided Methods

With improved computing power, virtual ligand screening (VLS) has increasingly become the most common cost-efficient method for screening compound libraries; it has expanded the chemical space of unavailable but synthetically feasible chemical entities. As a result, it is no surprise that virtual screening has become the primary method for identifying the most current SH2 domain inhibitors. There are two major types of virtual screening methodologies: ligand-based to target proteins of unknown structure, and structure-based methods for proteins with known structures. These two methodologies are often applied in the same lead discovery workflow, often in combination with molecular dynamics (MD). The availability of 3D structures of the SH2 domain has paved the way for the application of structure-based methods in the discovery of SH2 domain inhibitors. Standard approaches for structure-based methods include computational methods such as molecular docking, homology modeling, pharmacophore modeling, and MD simulation.

MD simulations are valuable for discovering new interactions by offering atomic-level insights into how weak-binding fragments interact, which can improve ligand-binding affinity. Through this approach, Greisman et al. were able to discover and validate the binding positions of allosteric fragment hits to protein tyrosine phosphatase 1b [79]. These in silico approaches have also been applied to the kinase family of proteins. For example, Samanta et al. recently used a combination of ligand-based VS and a combination of e-pharmacophore modeling, virtual screening, ensemble docking, and core hopping to identify six hits predicted to have high affinity for the SH2 domain of p56lck tyrosine kinase, five of which had favorable ADMET property prediction [80].

There is still a need to expand and focus in silico drug discovery campaigns to other non-enzymatic SH2 domain families, such as adaptor and docking proteins that account for over 30% of SH2 domain proteins and play a major role in the pathogenesis of many disease conditions. Breakthroughs in protein structure prediction have led to the development of new deep learning methodologies with unprecedented atomic-level predictive power of 3D protein structures.

## 7. Focus on Targeting SH2-Containing Transcription Factors

Among all the SH2-containing protein families, transcription factors such as STAT3 have attracted special attention as therapeutic targets due to their prominent role in many maladaptive responses and the scientific challenge, to this point, in targeting them. Dysregulation of STAT3, for example, has been implicated in a wide range of disease conditions, including chronic inflammation, fibrosis, and many cancers [85]. There are many studies that have used homology modeling, docking, and MD to screen for STAT3 inhibitors with some success.

Our efforts to identify STAT3 inhibitors began with the crystal structure of the EGFR Y1068 pY-peptide ligand bound to GRB2 [55]; this pY-ligand also binds to STAT3 and does so with the highest affinity of any STAT3-binding pY-peptide [86]. In the GRB2-pY1068–peptide crystal structure, the peptide had a b-turn, forced by a Trp (W) 121 residue within the GRB2 pY-peptide-binding groove (see Figure 3B). Docking of the pY1068-peptide crystal structure into the crystal structure of STAT3 monomer identified a W residue in an analogous position within STAT3’s pY-peptide-binding groove and also identified residues that are critical to the binding of the pY+3 residue, typically Q, that are distinct from those identified within the crystal structure of the STAT3 homodimer. Of note, this structure was not well resolved in regions showing reciprocal SH2/pY-peptide binding. We performed VLS by docking 920,000 compounds from several commercial libraries into a 10 angstrom cube containing all residues critical for pY-peptide binding [87]. The screen resulted in the discovery of three promising probes. A subsequent 2D fingerprint screen of 1.4 million compounds using the most potent probe (C188) followed by 3D pharmacophore sorting identified several potent second generation STAT3 probes, of which one, C188-9 or TTI-101, has subsequently demonstrated safety and a promising signal of efficacy in a phase 1 trial of advanced solid tumors [88] and is undergoing phase 2 testing for the treatment of hepatocarcinoma carcinoma and idiopathic pulmonary fibrosis [89]. In another example, Kong et. al. identified small-molecule STAT3 inhibitors using MD simulations to generate an averaged structure of the induced conformation of the STAT3 SH2 domain complexed to peptidomimetic CJ-887. This structure was then used to screen 110,000 compounds within the SPEC database, which identified two highly effective compounds with favorable drug-like properties. Inhibitors for STAT5, another SH2-containing transcription factor, have been identified using a similar computer-aided approach; the hits identified have shown efficacy against STAT5-driven cancers in preclinical models [90].

## 8. Conclusions

The SH2 domain is the major protein module that controls almost all the tyrosine kinase signaling networks and forms part of functionally diverse modular proteins that are expressed throughout the eukaryotic genome. Proteins containing SH2 domains have been implicated in many human diseases, attracting significant interest as both therapeutic targets and as tools for understanding fundamental biological and pathophysiological processes. Understanding the mechanisms and biological significance of the protein–protein interaction networks that are regulated by SH2 domain proteins offers potential for discovering novel therapeutic targets and developing promising pharmacological agents for a broad spectrum of diseases. Nearly four decades after their discovery, significant advances in technology and studies of SH2–ligand interactions still have not fully unlocked the clinical potential of targeting SH2 domain-containing proteins. This article focuses on the current trends in drug discovery for proteins containing SH2 domains and details the methods for targeting them. These methods include the design of peptidomimetics and small-molecule inhibitors (orthosteric, allosteric, and proximity-inducing bifunctional groups such as PROTACs) that incorporate structural models, including those generated by deep learning structure prediction tools like AlphaFold2. These approaches are likely to yield more potent and selective compounds than those identified by docking studies that are based solely on experimentally determined structures [91]; they offer transformative potential that will expand the chemical space of molecules that target SH2-domain-containing proteins.

## Figures and Tables

**Table 1:** Groups of proteins containing SH2 domains. (Those marked bold are proteins with SH2 domains solved experimentally).

Function	Protein
**Enzymes**	**ABL1, ABL2, CBL, CSK, MATK, FER, JAK2, PIK3R2, PLCG1, PTPN6, PTPN11, SOCS2, SOCS4, SOCS6, SRC, FYN, LCK, HCK, ZAP70, SYK, BMX, BTK, TXK,** FPS, FRK, BRK, SRMS, TYK2, JAK1, JAK3, PIK3R3, PLCG2, SHIP1, SHIP2, SOCS1, SOCS3, SOCS5, SOCS7, CISH, FGR, YES, BLK, TEC, ITK
**Regulator (GTPase activity activator)**	**CHN1**, **CHN2, RASA1, VAV1, VAV2,** RIN1, RIN2, RIN3, VAV3
**Adaptor proteins**	**CRK, CRKL, GRB2, GRB7, GRB10, GRB14, NCK1, NCK2, SH2D1A, HSH2D**, **SH2D1B**, DAPP1, GADS GRAP, APS, LNK, SH2B, SH2D2A, SH2D7, SH2D3A SH2D3C, BCAR3, SH2D4A, SH2D4B, SH2D5, SLAP, SLAP2, BLNK, LCP2 CLNK, LCP2, CLNK
**Docking proteins**	**BRDG1, SHC1, SH3BP2**, SHB, SHD, SHE, SHF, SHC2, SHC3, SHC4, BKS
**Transcription factor**	**STAT1, STAT2, STAT3,** STAT4, STAT5, **STAT5B, STAT6,** SUPT6H
**Cytoskeletal protein**	TNS1, TENS2, TNS3, TNS4

**Table 3:** SH2 domain-containing proteins currently known to facilitate condensate formation.

Condensate Complex	Role	SH2-Containing Proteins	Ref
** FGFR2:SHP2:PLCγ1 **	Increased activity of RTK Signaling	SHP2, PLCγ1	[13]
**LAT-GRB2-SOS1**	The ligand binding/T-cell activation and phosphorylation.	ZAP70, LCK, GRB2, PLCγ1	[14]
** N-WASP–NCK **	T-cell signaling	NCK	[15]
**SLP65, CIN85**	B-cell signaling	SLP65	[16]

**Table 4:** Drugs with known structures that target the SH2 domain are currently in clinical development. (For a comprehensive list of STAT3 inhibitors, see [78]).

Target	Drug	Targeting Mechanism	Phase	Identifier
STAT3	TTI-101 (C188-9)	STAT3 SH2 domain	I (Completed) II (Liver cancer) II (Lung fibrosis)	NCT03195699 NCT05440708 NCT05671835
STAT3	WP1066	STAT3/JAK2	I/II	NCT04334863
STAT3	Napabucasin (BBI608 or GB201)	STAT3 SH2 domain	II/III	NCT03721744
STAT3	DSP-0337	STAT3 SH2 domain	I	NCT03416816
STAT3	SC-43	STAT3/SHP-1	I/II	NCT04733521
STAT3	Silibinin	STAT3 SH2 domain	Not applicable	NCT05689619
STAT3	YY201 (YY002)	STAT3 SH2 domain	I	NCT06225856
STAT3	KT-333	STAT3 degrader	I	NCT05225584
Shp2	TNO155	N-SH2/C-SH2/PTP interface		
Shp	JAB-3312	“latch” N-SH2/PTP	I/II/III	NCT05288205 NCT06416410 CTR20241931
